# Infiltrating Immune Cells in Gastric Cancer: A Novel Predicting Model for Prognosis

**DOI:** 10.7150/jca.51079

**Published:** 2021-01-01

**Authors:** Wenjie Li, Mengting Li, Haizhou Wang, Yanan Peng, Shouquan Dong, Yuanyuan Lu, Fan Wang, Fei Xu, Lan Liu, Qiu Zhao

**Affiliations:** 1 Department of Gastroenterology, Zhongnan Hospital of Wuhan University, Wuhan, China.; 2 Hubei Clinical Center and Key Lab of Intestinal and Colorectal Diseases, Wuhan, China.

**Keywords:** gastric cancer, TIICs, immune risk score model, prognosis, multivariate cox regression

## Abstract

**Objective**: Immune cells infiltrating has been proved to be associated with prognosis in gastric cancer (GC) by studies. This study aims to explore the prognosis value of infiltrating immune cells in gastric cancer.

**Methods:** In our study, the CIBERSORT algorithm was used to calculate the fraction of 22 tumor-infiltrating immune cells (TIIC) in 100 normal and 300 tumor samples from the GEO cohort and 30 normal and 344 tumor samples from the TCGA cohort. Univariate and multivariate Cox regression were used to construct an immune risk score model. Multivariate cox regression was also used to validate whether our risk score model could predict prognosis in GC independently. Furthermore, the model was validated in different patient subgroups to test its independence. *P<*0.05 was considered statistically significant.

**Results:** The results showed that the fraction of 3 immune cells increased in tumor tissues compared with normal tissues in both the GEO and TCGA cohort. Univariate cox regression analysis showed four cells significantly correlated with survival rate in GC (*P<*0.05). The immune risk score model was constructed based on the four cells through multivariate cox regression and further validated. The KM survival curve suggested that patients with high risk had poor prognosis than patients with low risk (*P<*0.05). ROC curve indicated the model was reliable (AUC= 0.67 in the GEO cohort, AUC = 0.65 in the TCGA cohort). Furthermore, multivariate Cox regression showed the model was an independent factor for overall survival predicting in GC (hazard ratio (HR) = 2.35, 95% confidence interval (CI) = 1.63~3.40 in the GEO cohort, HR = 2.87, 95% CI = 1.94~4.25 in the TCGA cohort). Finally, we validated the model in patient subgroups by the KM survival curve.

**Conclusion:** In summary, tumor-infiltrating immune cells play an essential role in GC progression and affect the outcome of GC patients. The immune risk score can predict overall survival for GC independently, and high immune risk score is associated with poor prognosis.

## Introduction

Human gastric cancer is common cancer and the third leading cause of cancer-related death based on the Global Cancer Statistics 2018 1. Gastric cancer is a heterogeneous disease, and the outcome may change significantly even for patients with similar clinical characteristics and treatment options 2, 3. The current method of staging by pathology and tumor-node-metastasis (TNM) staging system is critical for choosing appropriate treatment 4, but it is not enough for prognostication and risk stratification 2, 3. Therefore, it is necessary to identify novel biomarkers providing high predicting values and raising the prognostication.

During the complex development of human tumors, six biological capabilities are recognized as the hallmarks of cancer 5. Tumors recruit a large number of normal cells, including immune cells, vessels, and fibroblasts to constitute the tumor microenvironment in which immune cells can foster multiple biological capabilities 6. It has been revealed that many solid tumors are associated with immune cell infiltration in the tumor immune microenvironment (TIME) which affects therapy efficacy and overall survival to no small extent 7-9. A study revealed that high T-cell subsets density in gastric cancer was associated with prolonged survival 10. What is more, it was thought that natural killer cells and dendritic cells were promising targets of immunity therapy for gastric cancer 11, 12. Hence, research on immune cells is critical for patient stratification and therapy selection.

In this study, we used CIBERSORT to calculate the composition of infiltrating immune cells in Gastric cancer and investigate the correlation between immune cells and overall survival and stages. A risk score model was constructed by multivariate cox regression to predict the overall survival of Gastric cancer, and the model was validated using clinical characteristics by univariate cox regression and multivariate cox regression.

## Methods

### Patient Cohort and Data Preparation

The training cohort is the ACRG cohort included 300 patients from the Asian Cancer Research Group study (GEO, https://www.ncbi.nlm.nih.gov/geo/) (GSE66229), with Affymetrix Human Genome U133 Plus 2.0 Array expression series matrix files and GPL570 platform. The testing cohort contained 348 patients from the “TCGA-STAD” project and the corresponding level-3 gene expression data were obtained from the Genomic Data Commons (https://portal.gdc.cancer.gov). The expression data was the HTSeq-FPKM type. For datasets in the GEO cohort, the robust multiarray average algorithm was applied for background correction and quartile normalization. For gene symbols with multiple probes, the average value was calculated as expression level. For both cohorts, only patients with available expression profiles, clinical, pathologic, and survival data were included for analyses.

### Evaluation of Infiltrated Immune Cells

The CIBERSORT algorithm was used to calculate the proportion of immune cells in gastric tumor tissues and normal tissues, with reference to LM22 gene signature 13. The CIBERSORT algorithm is an analytical tool, containing 547 marker genes' expression signature matrix for calculating the fractions of infiltrated immune cell composition. LM22 defines 22 subtypes of immune cells referring to the annotated gene signature matrix, downloaded from the CIBERSORT website portal (https:// cibersort.stanford.edu/).

The 22 immune cells contain two subtypes of B cells, seven subtypes of T cells, two subtypes of NK cells, three subtypes of Macrophages, Monocytes, Dendritic cells resting, Dendritic cells activated, Mast cells resting, Mast cells activated, Eosinophils and Neutrophils. Samples with CIBERSORT *p*-value < 0.05 were kept for the following analysis.

### Identification of Immune Cells correlated with Clinical Information

Only samples with CIBERSORT *p*-value < 0.05 were selected for clinical analysis in both TCGA and GEO cohort. The correlations between immune cells and overall survival and tumor pathologic stages were analyzed after merging selected immune cell expression matrix with the clinical information matrix. The cells with *p*-value <0.05 were considered as statistically significant.

### Immune Risk Score Model Construction

Univariate Cox proportional hazards regression was conducted in the training cohort (the GEO cohort) using the infiltrated immune cells, and cells with *p*-value <0.5 were included for subsequent analysis. “glmnet” R package 14 was used to construct the immune risk score model through multivariate Cox proportional hazards regression. Moreover, cells with *p*-value <0.05 were considered as independent prognostic indexes.

### Statistical Analysis

R 4.0.1 and appropriate packages were used to conduct all statistical analyses. The infiltrations of 22 immune cells were assessed by Wilcox test. Correlations among different types of immune cells were analyzed by “corrplot” R package. Kaplan-Meier survival curve was analyzed by “survival” R package and evaluated by log-rank test. The correlations between immune cells and pathologic stage and TNM stage were evaluated by Wilcox test. Time-dependent ROC curves were analyzed by “ROC” R package. All statistical tests were two-sided, and *p*-value <0.05 was considered statistically significant.

## Results

### Clinical Characteristics

**Figure 1:**
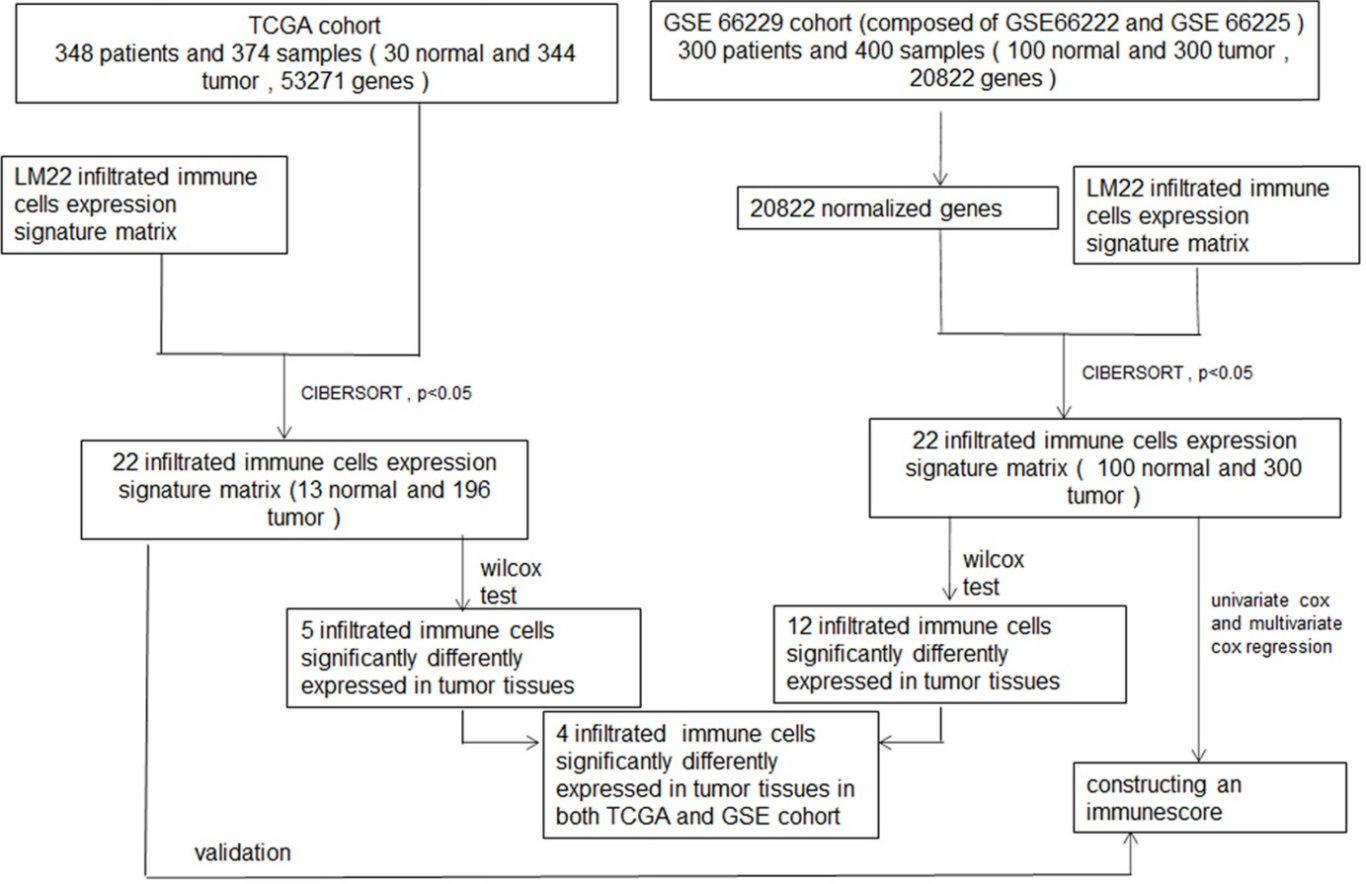
Flowchart of whole procedure including immune cells analysis and their correlations with clinical information, and immune score model construction of immune cells, and validation of the model.

The procedure for the study is shown in the flowchart in detail (**Figure 1**). Three hundred patients and 348 patients diagnosed with GC were included in the GEO cohort and TCGA cohort, respectively. GEO cohort included 199 (66.33%) male and 101 (33.67%) female, and the TCGA cohort included 110 (31.61%) male and 235 (67.53%) female. Rest detailed clinical characteristics including age, pathologic stage, and TNM stage of all patients of these two cohorts are shown in **Tables 1 & 2.**

### Identification of Differentially Expressed Immune Cells between Normal Tissue and Tumor Tissue

**Figure 2:**
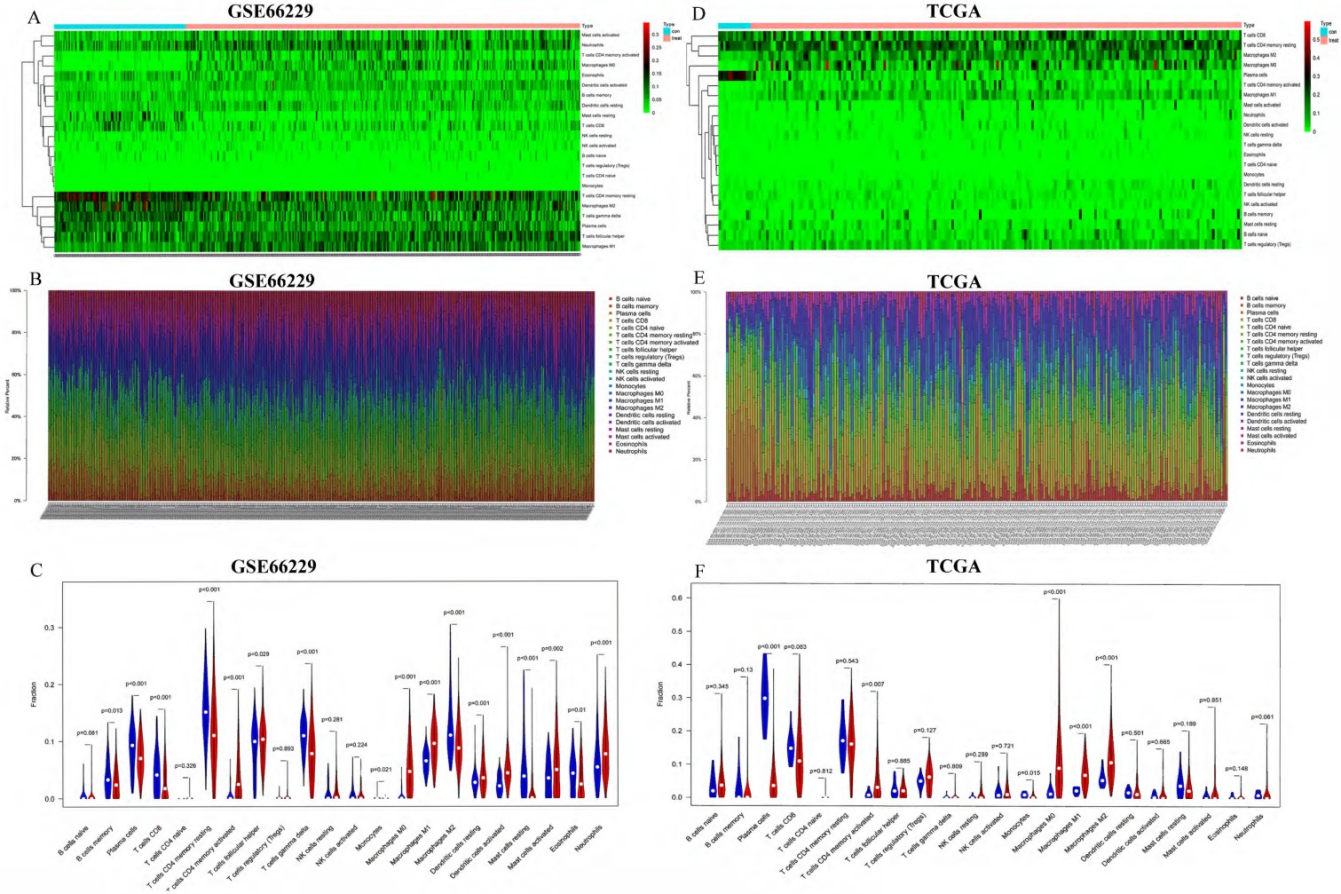
Analysis of TIICs in 100 normal and 300 tumor tissues in the GEO cohort and 13 normal and 196 tumor tissues in the TCGA cohort. (**A, D**) Heatmaps of TIICs in normal and tumor tissues from the GEO cohort and TCGA cohort, respectively. (**B, E**) Barplots of TIICs in normal and tumor tissues from the GEO cohort and TCGA cohort. (**C, F**) The different fraction of immune cells between normal and tumor tissues.

First, we studied the ratio of immune cell composition between tumors and normal tissues in the GEO and TCGA cohorts, respectively. In the GEO cohort, 100 normal and 300 tumor samples were qualified with CIBERSORT *p*-value <0.05. The composition of 22 infiltrating immune cells was analyzed. We performed heatmap and barplot analysis of foresaid immune cells to illustrate the differential composition of immune cells in different samples (**Figure 2A-B**). In **Figure 2C**, we showed that the formation of many immune cells was significantly different between normal tissue and GC tissue. T cells CD8, T cells CD4 memory resting, T cells CD4 memory activated, T cells follicular helper, T cells gamma delta, Macrophages M0, Macrophages M1, Dendritic cells resting, Dendritic cells activated, Mast cells activated, Eosinophils and Neutrophils were noticeably increased in GC tissues compared to normal tissues with *p*-value <0.05. In the TCGA cohort, 13 normal and 196 tumor samples were qualified with CIBERSORT *p*-value <0.05. The same as the GEO cohort, we analyzed the composition of 22 infiltrating immune cells. The composition of the immune cells in normal and tumor tissues was shown in **Figure 2D-2E.** The fractions of T cells CD4 memory activated, macrophages M0, macrophages M1 and macrophages M2 were significantly higher (*p*-value <0.05) in GC tissues than in normal tissues, shown in **Figure 2F.**

### Relationships between Immune Cells in GC Tissues and Correlation of Immune Cells with Clinical Features

**Figure 3:**
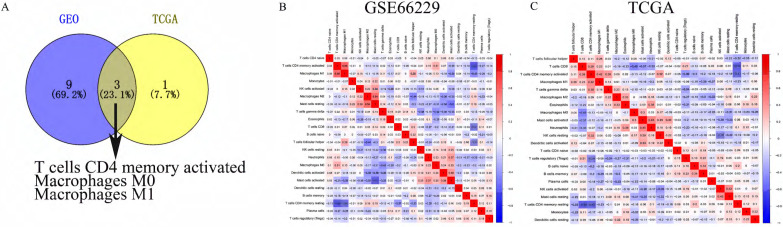
(**A**) The blue cycle represented immune cells increased in the GEO cohort. The yellow cycle represented immune cells increased in the TCGA cohort. Correlation of immune cells with each other in GEO (**B**) and TCGA (**C**).

To identify immune cells increased in both the GEO cohort and the TCGA cohort, we took the intersection of immune cells increased in both cohorts. As shown in **Figure 3A**, three immune cells (T cells CD4 memory activated, Macrophages M0 and Macrophages M1) were found higher in GC tissues than in normal tissues in both GEO and TCGA cohort. The relationships between different types of immune cells were analyzed by the “corrplot” package via Pearson correlation coefficient.

As shown in **Figure 3B**, T cells CD4 memory activated was positively related with Macrophages M1 and Mast cells resting with Correlation coefficient > 0.5. T cells CD4 memory activated was negatively correlated with T cells CD4 memory resting, and Mast cells activated was negatively correlated with Mast cells resting with Correlation coefficient < -0.5 in the GEO cohort. In the TCGA cohort (**Figure 3C**), T cells CD4 memory resting was negatively related with T cells CD8 and T cells CD4 memory activated with Correlation coefficient -0.49 and -0.47, respectively, which was partly consistent with the correlation of immune cells in the GEO cohort.

**Figure 4:**
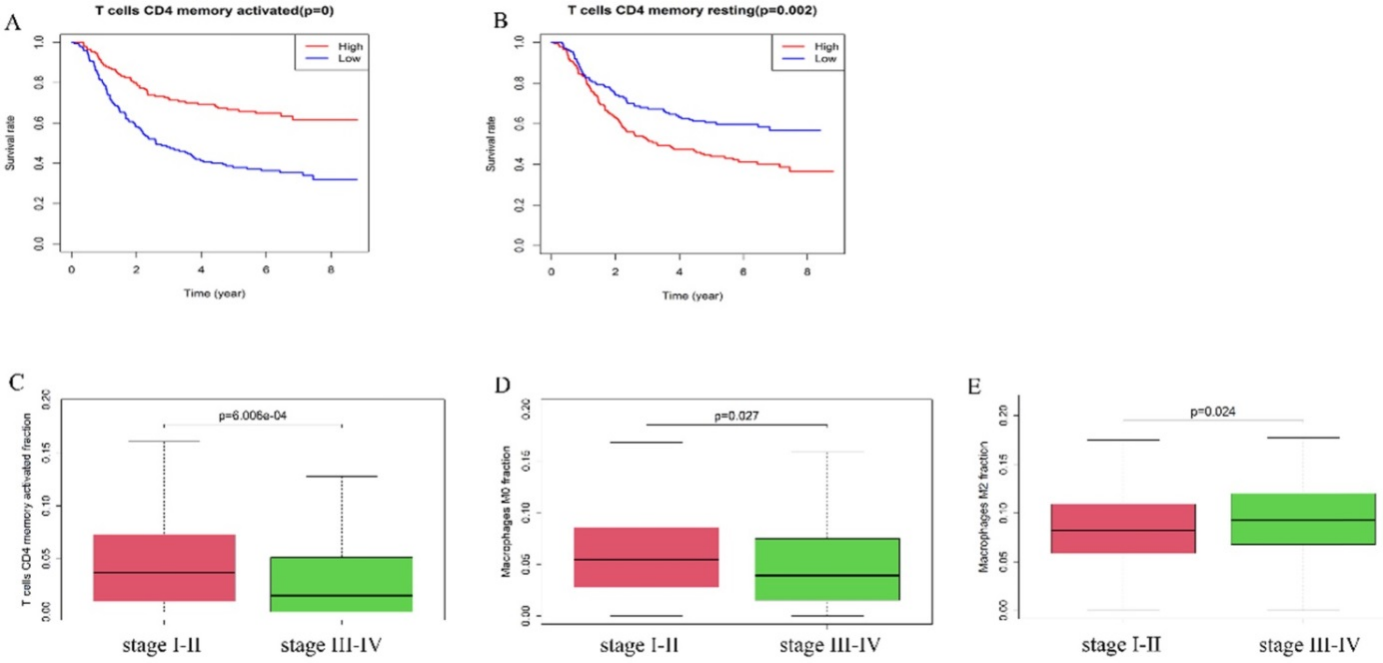
A high fraction of T cells CD4 memory activated (**A**) and a low fraction of T cells CD4 memory resting (**B**) was associated with better prognosis. (**C**) T cells CD4 memory activated and (**D**) Macrophages M0 were lower in stage III-IV than stage I-II. (**E**) Macrophages M2 was higher in stage III-IV than stage I-II.

Furthermore, we explored the correlation of immune cells with the overall survival and pathologic stage. In the GEO cohort, high fraction of T cells CD4 memory activated (*p*-value <0.01) was associated with better survival rate (**Figure 4A**), while a high fraction of T cells memory resting (*p*=0.002) was a risk factor (**Figure 4B**). The fraction of T cells CD4 memory activated (*P<*0.001) and Macrophages M0 (*p*=0.027) was higher in stage I-II tumors compared with stage III-IV tumors while the fraction of Macrophages M2 (*p*=0.024) was higher in stage III-IV tumors than in stage I-II tumors (**Figure 4C-E**).

### Construction and Validation of the Immune Risk Score Model

**Figure 5:**
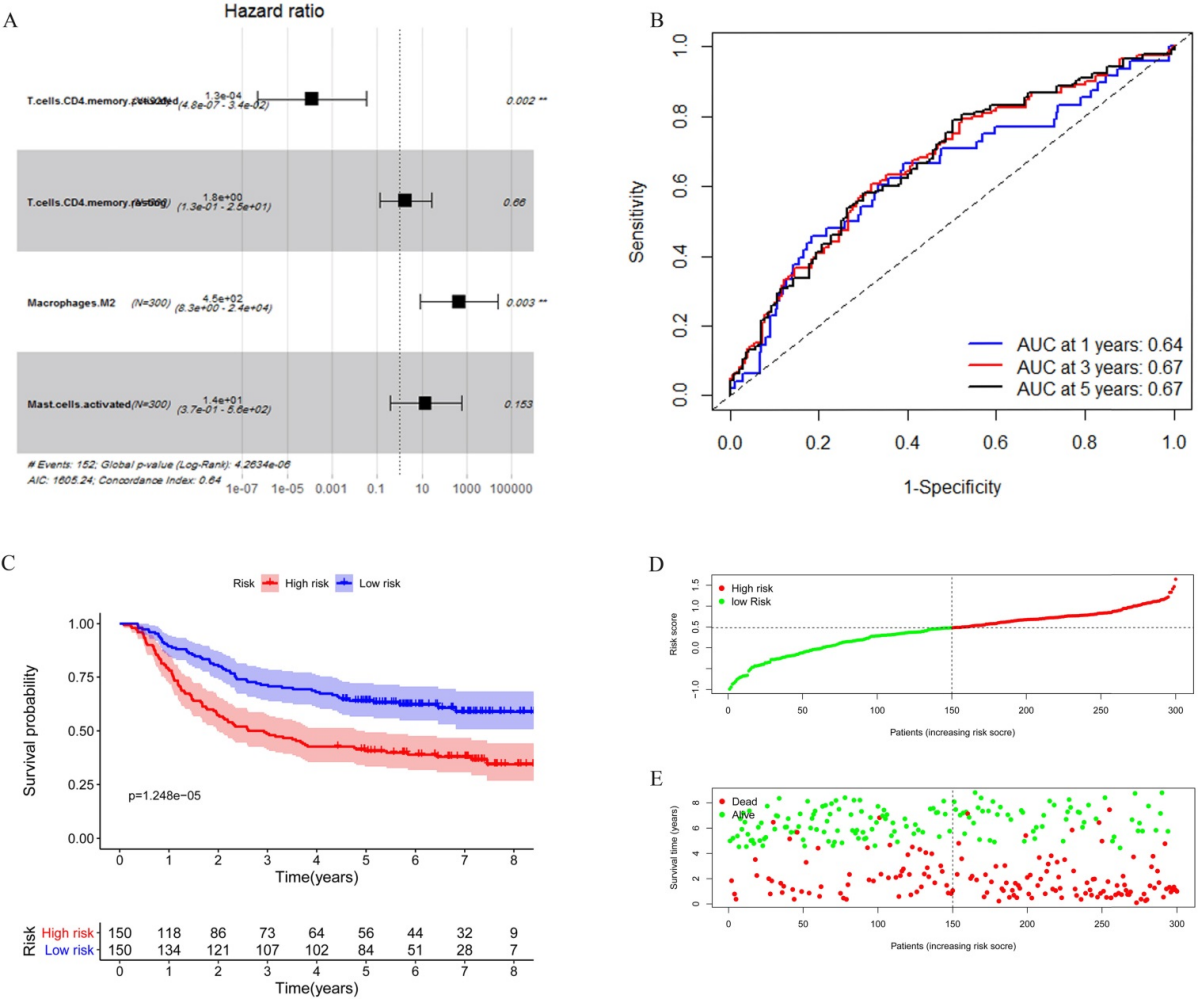
Construction of the immune risk score model. (**A**) T cells CD4 memory activated, T cells CD4 memory resting, Macrophages M2, and Mast cells activated were selected to construct an immune risk score model through multivariate cox regression in the GEO cohort. (**B**) ROC curve analysis of prognosis prediction by the model. (**C**) KM survival curve indicted that high-risk scores were associated with poor prognosis. (**D**) Distribution of the immune score of GC patients. (**E**) Distribution of patients' status.

Considering the number of samples, we chose the GEO cohort as the training set. Univariate Cox regression was applied to identify immune cells associated with the prognosis of GC patients. As shown in **Table 3**, T cells CD4 memory activated, T cells CD4 memory resting, Macrophages M2, and Mast cells activated were found associated with gastric cancer survival risk significantly with *p*-value <0.05. Moreover, these four immune cells were included for multivariate Cox regression analysis to construct an immune risk score model (**Figure 5A**) in which we could see that a high fraction of T cell CD4 memory activated was a potential protective factor. The risk score calculation formula was: risk score= (0.5500 * fraction of T cells CD4 memory resting) + (-8.8257 * fraction of T cells CD4 memory activated) + (5.8963 * fraction of Macrophages M2) + (2.5130 * fraction of Mast cells activated). The ROC curve showed that this model had a passable sensitivity and specificity in predicting overall survival. The AUC of one-year, three-year, and five-year survival was 0.64, 0.67, and 0.67, respectively (**Figure 5B**). Moreover, we calculated the risk score of each patient through this model, and the KM survival curve indicated that patients with high-risk scores had poor prognosis compared with patients with low-risk scores (**Figure 5C**). We ranked GC patients according to risk score (**Figure 5D**), and patients' status was presented in **Figure 5E.** We found that death occurred more in the high-risk group than in the low-risk group, which was consistent with our previous result.

**Figure 6:**
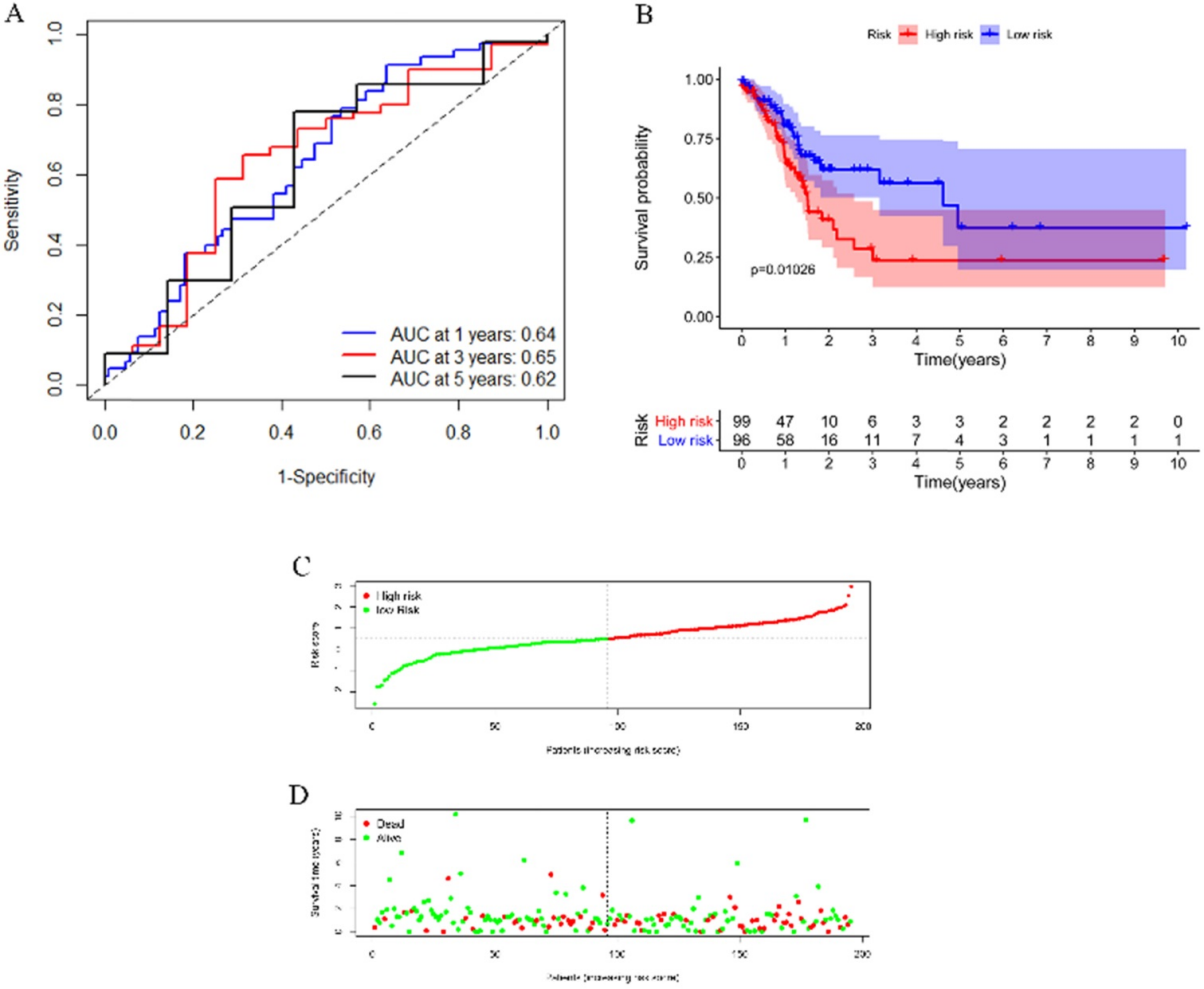
Validation of the model by the TCGA cohort. (**A**) ROC curve analysis of survival prediction by the model. (**B**) KM survival curve revealed that patients with high risk had a poor prognosis. (**C**) Distribution of the immune score of GC patients. (**D**) Distribution of status of GC patients.

To test the applicability of our model, we applied the model in the TCGA cohort for validation. Each patient in the TCGA cohort was assigned a risk score according to the risk score model. As shown in **Figure 6A**, this model still had a passable sensitivity and specificity in predicting overall survival. Low-risk patients had significantly better survival probability than high-risk patients, which was indicated by the KM survival plot (**Figure 6B**). The distribution of risk score and patients' survival status was presented in **Figure 6C-D.** Consistent with the training cohort, more deaths occurred in the high-risk group than in the low-risk group.

### The Immune Risk Score Model could predict the Prognosis of Gastric Cancer Independently

**Figure 7:**
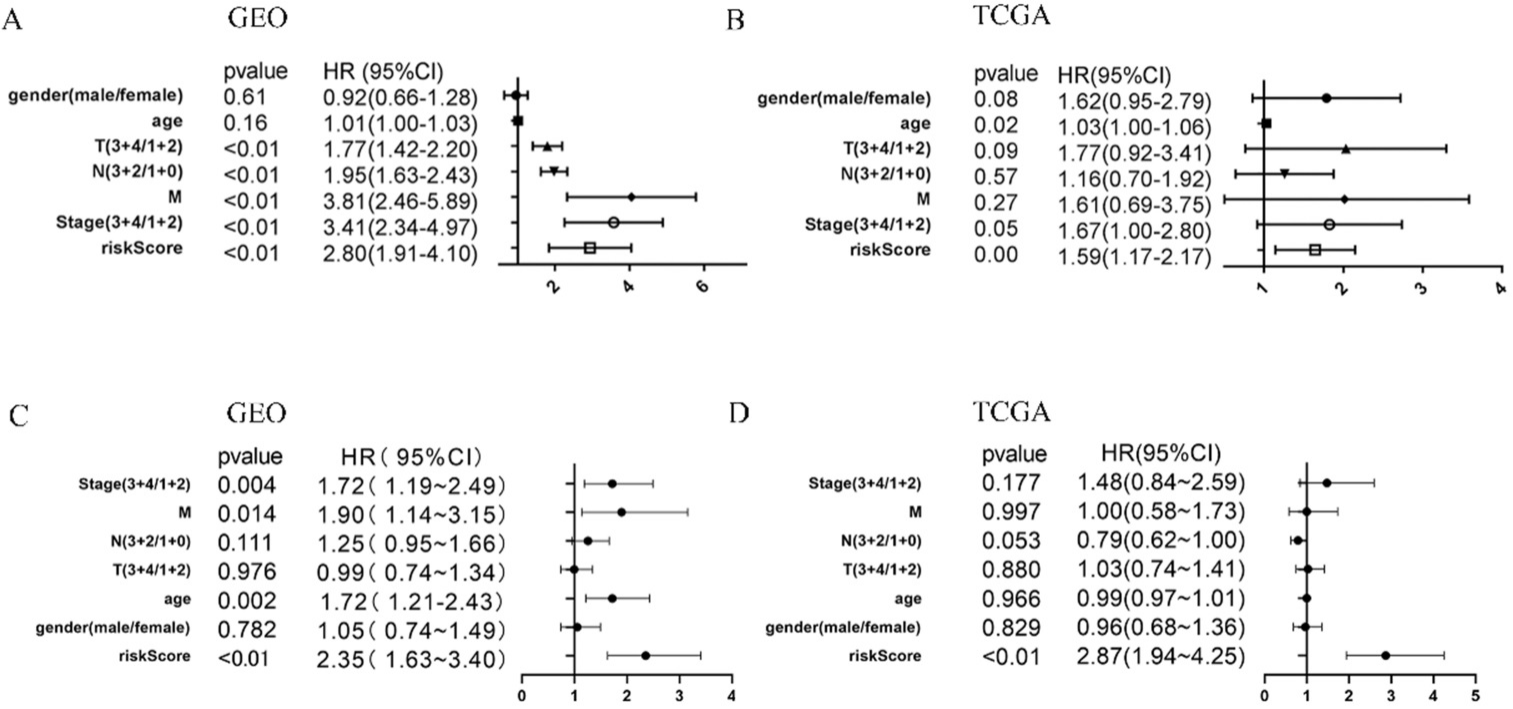
COX regression of clinical factors might affect prognosis. Univariate cox regression of seven factors in the GEO cohort (**A**) and TCGA cohort (**B**). multivariate cox regression of seven factors in the GEO cohort (**C**) and TCGA cohort (**D**).

**Figure 8:**
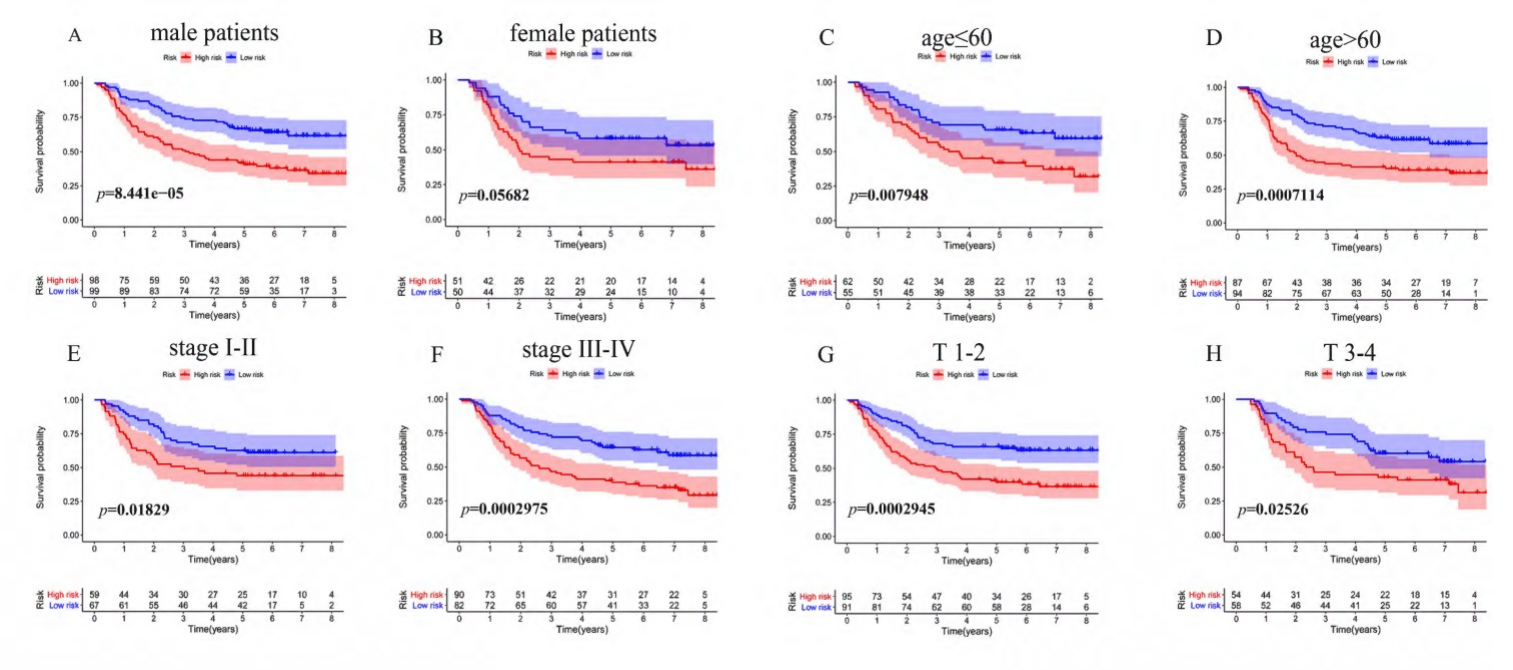
KM survival analysis of different patient subgroups in the GEO cohort. (**A**) male patients, (**B**) female patients, (**C**) patients with age ≤ 60, (**D**) patients with age >60, (**E**) patients with stage I-II GC, (**F**) patients with stage III-IV, (**G**) patients with T I-II, and (**H**) patients with T III-IV.

To determine whether the immune risk score model was independent of clinical parameters, we performed univariate cox and multivariate cox regression. In the GEO cohort (**Figure 7A**), high-risk score, T, N, M, and pathologic stage were related to poor prognosis while age and high-risk score were associated with poor prognosis in the TCGA cohort (**Figure 7B**). To identify factors that can predict prognosis independently, we applied multivariate Cox regression. As shown in **Figure 7C**, stages, TNM-M stage, age, and risk score were independent factors with *p*-value <0.05 in the GEO cohort, while in the TCGA cohort, only risk score could predict overall survival independently (**Figure 7D**). It proved that our model was an independent and reliable factor for estimating the prognosis of Gastric cancer. Furthermore, the risk score model was validated within patients subgroups divided by age, gender, stage, and T-stages, respectively, in the training cohort. Similarly, high-risk patients had poorer outcome compared with low-risk patients in the male (*P<*001), age <60 (*P<*0.01), age ≥60 (*P<*0.001), stage I-II (*p*=0.01859), stage III-IV (*P<*0.001), TI-II (*P<*0.001), and TIII-IV (*p*=0.02526) subgroups (**Figure 8A, 8C-H**). However, the high-risk patients in the female subgroup (*p*=0.05682) didn't significantly differ with low-risk patients in survival rate (**Figure 8B**). Together, these results indicated the reliability of our model.

## Discussion

Gastric cancer is a common cause of tumor-related death. Due to the heterogeneity of Gastric cancer, patients even with the same pathologic stage and TNM stage responded differently to similar therapy. Recently, immunotherapy based on blockade of immune checkpoints has obtained appreciable efficacy in GC. The infiltrating immune cells, especially T cells in tumors, play a vital role in recognizing and eradicating cancer cells, and cancer cells use multiple signaling pathways to inhibit the activity of T cell, thus suppressing tumor immunity 15. The therapy blocking PD-1/PD-L1 exerted a favorable response rate by reactivating the effector activity of infiltrating T cells in many cancers 16, 17. However, the cancer cells inhibit immune cells function by multiple mechanisms 18 for which immunotherapy does not suit each patient. Therefore, the identification of novel biomarkers is vital for stratifying patients and choosing appropriate therapy. Based on this purpose, we analyzed 22 infiltrating immune cells in Gastric cancer and constructed an immune risk score model using immune cells to predict overall survival in this study.

We performed the CIBERSORT algorithm to evaluate the composition fraction of immune cells in gastric cancer tissues and normal tissues. T cells CD4 memory activated, macrophages M0, and macrophages M1 were three immune cells increased in tumor tissues compared with normal tissues in both GEO and TCGA cohorts. Macrophages are one of the primary immune cells in tumor immune microenvironment and can function differently in response to microenvironmental signals 19, 20. Macrophages are versatile cells that can be polarized into pro-inflammatory macrophages M1 and immunosuppressive macrophages M2 under different chemokines stimulation 21. Both M1 and M2 participate in the tumor progression process, but M1 is a mainly protective factor by activating the production of toxic intermediates and reactive oxygen intermediates, while M2 is a risk factor for it promotes tumor progression and metastasis 22, 23. Macrophages M0 has similar but slightly weaker functions as M1. In our analysis, Macrophages had no significant relationship with overall survival, but significantly correlated with pathologic stages. In the GEO cohort, the correlations of Macrophages and stages were consistent with foresaid studies.

In our survival analysis, a high fraction of T cells CD4 memory activated was related to high survival possibility while a high fraction of T cells CD4 memory resting was associated with poor survival probability. T cells are able to recognize and eradicate cancer cells through multiple steps 15. It has been reported that polyfunctional CD8+ effector cytotoxic T cells (CTL) *in vivo* are critical for anti-tumor immunity 24. A study has shown that with the help of CD4 T cells, CTL polyfunctionality and anti-tumor effect both increased 25. Moreover, CD4 memory T cells display multifunctional and resistant cytokine production in response to stimulation 26. A study about bladder cancer also indicated that a high fraction of T cells CD4 memory activated was associated with better outcomes 27. Cancer cells can not only activate T cell responses but can also inhibit the activity of T cells through overexpression of checkpoint molecules, which is called immune tolerance 15. Besides, tumor cells can adjust the tumor immune microenvironment to facilitate the growth of tumor cells by expanding regulatory T-cells (Tregs) and other immune cells-regulatory cells 28, 29. Normally, the resting formation of immune cells is associated with the dormant immune microenvironment and poor prognosis. The current immunotherapies aimed to reactivate dormant anti-tumor immunity by targeting checkpoint molecules have achieved considerable progress 30. These results suggested that T cells CD4 memory activated could restrict the growth of cancer cells. In contrast, T cells CD4 memory resting might associate with poor prognosis partly because it correlates with immune tolerance.

Furthermore, univariate cox and multivariate cox regression were performed to construct an immune risk model using the 22 infiltrating immune cells. KM survival curve suggested that patients with high-risk scores had significantly poor survival rates than patients with low-risk scores in both TCGA and GEO cohorts (*P<*0.001 and p=0.01026, respectively). ROC curve demonstrated the reliability of the model in predicting overall survival (AUC at three years was 0.67 and 0.65 in the GEO cohort and the TCGA cohort, respectively). We also performed multivariate cox regression to validate our model as an independent factor for predicting overall survival of gastric cancer. Currently, immunotherapies based on blockade of immune checkpoints (such as CTLA-4 31, PD1, and PD-L1) receptors and their ligands have made considerable progress not only in Gastric cancer but also in colon, lung, and renal carcinoma.^16^ However, effective biomarkers for prognosis predicting and selecting patients sensitive to immunotherapy and can benefit from the therapy the best remains unclear. The efficacy of immunotherapy largely depends on the response of infiltrating immune cells in the tumor immune microenvironment 17, 18. Hence, discovering an immune biomarker for predicting prognosis and choosing therapy is of great importance.

The role of infiltrating immune cells in gastric cancer has been explored in several previous studies. Liu K et al. explored the distribution and density of tumor-infiltrating immune cells in gastric cancer through immunostaining and indicated that immune cells were significantly associated with the prognosis of GC patients 32. Wang JT et al. revealed that IL17A+ cells infiltration improved anti-tumor contexture and response to immunotherapy in GC 33. Li L et al. constructed an immune score model by tumor-infiltrating immune cells; however, they only validated the nomogram model instead of the score model by external datasets 34. In our study, we constructed an immune score model with reliability and validated it with an external dataset, which is our strength. Some studies investigated the relationship between immune checkpoint molecules and immune cells and their correlation with overall survival. Wang et al. reported that PD-L1 expression and CD8 T cell infiltration were associated with better outcomes in advanced GC 35. Wang M et al. identified SUPV3L1 and SLC22A17 as two genes that could affect the immune cells and prognosis in GC 36. Zhou Y et al. found that Fatty acid synthase (FASN) was associated with immune cells and the prognosis of Gastric cancer patients 37. All these studies provided a new comprehension of how Gastric cancer progressed and potential therapies targets. However, the shortcoming of these studies is that they focused on only one gene or one cell and neglected that tumors comprise complex biological processes and varieties of cells. It should be noted that we considered four immune cells when constructing the immune risk score model. Besides, the model was constructed in the GEO cohort in which data was from Asian people but validated in the TCGA cohort in which data was from people without specific ethnicities. Therefore, we thought our model might be able to extrapolate to different races. There certainly are limitations to our study. The major limitation of our study is that we didn't validate our results by experiments or our own clinical specimens.

## Conclusion

In summary, the fraction of immune cells is correlated with survival in GC. Our model is a reliable and independent factor for predicting prognosis. With the development of sequencing technology, we think our model has great potential in clinical practice. The model may play an essential role in patient stratification and therapy.

## Figures and Tables

**Table 1:** The characteristics of patients in the GEO cohort

Variables	Case, N (%)
**Age at diagnosis**	
≤60	117 (39.00%)
>60	183 (61.00%)
**Gender**	
Male	199 (66.33%)
Female	101 (33.67%)
**Pathological-Stage**	
I	30 (10.00%)
II	96 (32.00%)
III	95 (31.67%)
IV	77 (25.67%)
NA	2 (0.67%)
**TNM-T**	
T1	0 (0.00%)
T2	186 (62.00%)
T3	91 (30.33%)
T4	21 (7.00%)
NA	2 (0.67%)
**TNM-N**	
N0	38 (12.67%)
N1	131 (43.67%)
N2	80 (26.67%)
N3	51 (17.00%)
**TNM-M**	
M0	273 (91.00%)
M1	27 (9.00%)

**Table 2:** The characteristics of patients in the TCGA cohort

Variables	Case, N (%)
**Age at diagnosis**	110 (31.61%)
≤60	235 (67.53%)
>60	2 (0.57%)
NA	
**Gender**	
Male	218 (62.64%)
Female	129 (39.94%)
**Pathological-Stage**	
I	52 (14.94%)
II	103 (19.60%)
III	135 (38.79%)
IV	35 (10.05%)
NA	22 (6.32%)
**TNM-T**	
T1	20 (5.75%)
T2	78 (22.41%)
T3	156 (44.83%)
T4	85 (24.43%)
NA	8 (2.30%)
**TNM-N**	
N0	104 (29.89%)
N1	91 (26.15%)
N2	71 (20.4 0%)
N3	65 (18.68%)
NA	16 (4.60%)
**TNM-M**	
M0	307 (88.22%)
M1	24 (6.90%)
NA	16 (4.60%)

**Table 3:** Univariate Cox Regression Analysis for 22 Immune Cells in Gastric cancer Patients

ID	HR	HR.95L	HR.95H	pvalue
T cells CD4 memory activated	0.000515	1.56E-05	0.017058	2.24E-05
T cells CD4 memory resting	12.02513	2.133142	67.78905	0.004824
Macrophages M2	52.22081	2.458491	1109.222	0.011184
Mast cells activated	11.88592	1.059103	133.3913	0.044802
Plasma cells	0.030911	0.000776	1.231519	0.064429
Macrophages M1	0.053303	0.001711	1.6603	0.094723
Dendritic cells resting	11.73588	0.205767	669.3522	0.232616
T cells gamma delta	4.242797	0.333383	53.9959	0.265462
Neutrophils	0.214906	0.012666	3.646489	0.287165
Eosinophils	0.142002	0.003614	5.579845	0.297358
Macrophages M0	0.213325	0.010996	4.138529	0.307179
T cells follicular helper	0.254205	0.012217	5.289503	0.376491
NK cells resting	0.053226	6.20E-05	45.67551	0.394715
T cells CD4 naive	1.51E-08	1.17E-26	1.96E+10	0.397427
Dendritic cells activated	0.243218	0.001937	30.53197	0.566374
T cells regulatory (Tregs)	0.003249	8.44E-12	1250128	0.569996
B cells naive	8.782117	0.004589	16806.77	0.573078
NK cells activated	6.86765	0.005365	8791.848	0.597617
Mast cells resting	1.576252	0.013688	181.5206	0.850947
T cells CD8	0.743425	0.020419	27.0669	0.87158
B cells memory	0.874211	0.010045	76.08398	0.952957
Monocytes	0	0	Inf	0.994677
